# Oral healthcare of preschool children in Trinidad: a qualitative study of parents and caregivers

**DOI:** 10.1186/1472-6831-12-27

**Published:** 2012-08-03

**Authors:** Rahul Naidu, June Nunn, Maarit Forde

**Affiliations:** 1 Senior Lecturer, Community Dentistry, Faculty of Medical Sciences, The University of the West Indies, Port of Spain, Trinidad and Tobago; 2 Special Care Dentistry, Dean, School of Dental Sciences, Dublin Dental University Hospital, Lincoln Place, Dublin 2, Ireland; 3 Lecturer Department of Liberal Arts, The University of the West Indies, Port of Spain, Trinidad and Tobago

**Keywords:** Preschool children, Oral health, Parents, Caregivers, Qualitative, Focus groups, West Indies

## Abstract

**Background:**

Little is known about oral health in early childhood in the West Indies or the views and experiences of caregivers about preventive oral care and dental attendance The aims of this study were to explore and understand parents and caregivers’ experience of oral healthcare for their preschool aged children and how, within their own social context, this may have shaped their oral health attitudes and behaviours. These data can be used to inform oral health promotion strategies for this age group.

**Method:**

After ethical approval, a qualitative study was undertaken using a focus group approach with a purposive sample of parents and caregivers of preschool children in central Trinidad.

Group discussions were initiated by use of a topic guide. Audio recording and field notes from the three focus groups, with a total of 18 participants, were transcribed and analysed using a thematic approach.

**Results:**

Despite some ambivalence toward the importance of the primary teeth, the role of fluoride and confusion about when to take a child for their first dental visit, most participants understood the need to ensure good oral hygiene and dietary habits for their child. Problems expressed included, overcoming their own negative experiences of dentistry, which along with finding affordable and suitable dental clinics, affected their attitude to taking their child for a dental visit. There was difficulty in establishing good brushing routines and controlling sweet snacking in the face of many other responsibilities at home. Lack of availability of paediatric dental services locally and information on oral health care were also highlighted. Many expressed a need for more contact with dental professionals in non-clinic settings, for oral health care advice and guidance.

**Conclusion:**

Parents and caregivers in this qualitative study showed generally positive attitudes towards oral health but appear to have encountered several barriers and challenges to achieving ideal preventive care for their child, with respect to healthy diet, good oral hygiene and dental attendance. Oral health promotion should include effective dissemination of oral health information, more practical health advice and greater access to dental care for families with preschool children.

## Background

### Early childhood oral health

Poor oral health in early childhood is one of the most serious and costly health conditions in young children [1]. The main concern is that of decay in the primary dentition. Early childhood caries (ECC), decayed teeth in children under 6 years of age, is a multi-factorial childhood disease with sociocultural and socioeconomic determinants [2]. Untreated ECC and its more rampant subtype Severe Early Childhood Caries (S-ECC) are associated with negative health outcomes. These include, poor feeding and eating, increased irritability due to pain and discomfort, reduced weight gain, slowed cognitive development and poorer quality of life [3-7]. Furthermore invasive treatment for caries in preschool children may be distressing for the child and family. Internationally prevalence of ECC has been reported to range from 6-90%, with most developed countries in the lower end and most developing countries in the middle to higher end of this range.

### Dental services and children’s oral health in Trinidad and Tobago

Approximately 300 registered dentists practice in the island giving a dentist population ratio of about 1: 4,000. Most work privately in practices generally clustered in urban areas. Free dental care is available in the sector for children and adults in Regional Health Centres mainly for emergency care and exodontias, Currently 20 dentists work in the government sector supported by approximately 40 dental nurses (the equivalent of dental therapists in the UK). These dental nurses were trained using the New-Zealand model in the 1970’s and provide the mainstay of dentistry in the public sector for children up to 12 years of age. Paediatric dental services (emergency and routine care) are also available at the University dental clinic, for children up to the age of 16 years.

There is currently no published epidemiological data on preschool children’s oral health in Trinidad, however, the available information about oral health of children in this age group, suggests that ECC may be a problem in the region [8,9]. Also, the persistence of untreated caries in the primary dentition of school-aged children can be considered a public health problem, as a national survey in Trinidad reported almost two thirds of 6–8 year-olds had caries experience [10] and acute problems arising from decayed primary teeth were the most frequent cause of emergency dental visits in a dental hospital clinic [11]. Risk models have indicated that decayed primary teeth presenting in the primary school age indicate these children were at high risk for caries during their pre-school years [12].

### Role of parents and caregivers

In their conceptual model Fisher-Owens *et al.*[13], recognized multilevel influences on children’s oral health at the individual, family and community levels. These family level influences are mediated mainly through parents and caregivers with whom preschool children spend most of their time. During this period of primary socialization, routine dietary and health behaviors being established are directly and indirectly influenced by the oral health knowledge, attitudes, beliefs and practices of their parents and caregivers [14]. The influence of community level cultural factors and health-beliefs was also highlighted in the model proposed by Adair *et al.*[15]. Oral health knowledge and self-efficacy among mothers of preschool children are also important influences on oral health habits and routines in the home [16]. Parents and caregivers of preschool children attending a dental hospital clinic in Trinidad had inaccurate factual knowledge and low awareness of preventive care [17], indicating the need for a more in-depth understanding of their health beliefs and practices with respect to their children’s oral health care.

### Qualitative research

Qualitative research can be considered the study of human actions and activities or a way of witnessing human events in the context in which they occur [18]. By the use of a ‘naturalistic approach’, qualitative research attempts to study phenomena in a real-world setting [19,20].

Until relatively recently there have been few studies using qualitative methods to investigate oral health in infants and young children. In-depth interviews with parents of Finnish preschool children showed that significant barriers to dental care were the lack of oral health information, difficulties in making oral care part of the daily routine and finding time to make dental appointments. [21]. Dental and non-dental healthcare professionals in a focus group study revealed that they believed early childhood caries was an important problem which could cause significant pain and long term problems for children, such as low self esteem and the need complex orthodontic care [22].

Lack of awareness of the importance of primary teeth was a common finding in a focus group study with parents and caregivers in a multiethnic community in the US [23]. Qualitative studies of low-income families in Head Start programs in the US report that parents often saw the primary dentition as temporary teeth whose condition had little bearing on the future dentition [24] and though aware of some of the problems that could arise felt that general life demands made oral care a low priority [24]. Daly *et al.*[25] found that parents in their study (white mothers attending a Sure-Start program in the UK), viewed the primary dentition as important and were keen to establish good oral health habits. These reports suggest that social and cultural factors play a part in parental attitudes, as dental attendance for preventive dental care was not seen as necessary, rather only if problems arose.

Even where general knowledge and awareness was found to be good, a focus group study with pregnant mothers found that several had misconceptions about oral health such as the role of fluoride and integrity of the teeth during pregnancy [26]. Another common issue arising in these studies was the lack of availability and access to information on dental health for young children and specific advice on how to effectively translate this information into daily routines [25].

No qualitative investigation of the oral health of preschool children has been reported in the Caribbean. Research using a qualitative approach may identify specific oral health concerns among this population group and identify opportunities for oral health promotion and oral health policy development.

The aim of this study was to therefore gain understanding of parents’ and caregivers’ influence on preschool children’s oral health in a Trinidadian community, in the West Indies. In particular, to explore and understand parents’ and caregivers’ experiences of oral health and oral healthcare and how, within their own social context, this may have shaped their oral health attitudes and behaviours. Such information can help to guide the development of appropriate oral health promotion strategies to prevent ECC.

### Objectives

To explore the oral health beliefs of parents / caregivers of preschool children.

To describe parent / caregiver experiences of accessing dental care for themselves and their preschool children.

To explore parent / caregiver attitudes and behaviours toward oral care for preschool children and opportunities for oral health promotion to prevent ECC.

## Method

Focus groups were chosen as the qualitative research methodology for this investigation. This method was selected as it was considered to be the appropriate research design to provide in-depth understanding of the issues under investigation and be an efficient means to attain such data with respect to available time and resources.

A focus group session can be considered an unstructured interview with a group of people who are encouraged to interact with each other and the facilitator [27]. The session uses group dynamics to stimulate discussion, gain insights and generate ideas to explore a chosen topic in depth. They are able to produce a large amount of information in a relatively short space of time. Kitzinger [28] states that “focus groups can help people explore their views and generate questions in ways they would find difficult in face-to-face interviews and when group dynamics work well the participants work alongside the researcher, taking the research in new and often unexpected directions.” They have been found to work particularly well for groups discussing health priorities [27]. As a data collection technique focus groups are particularly sensitive to cultural variables by enabling analysis of shared identities and common knowledge operating within the group along with humor, consensus, and dissent used in their narratives [28].

### Sampling and recruitment

A non-probabilistic, purposive sampling method was used to recruit subjects to the study. The aim of this method was to obtain a sample representative of parents and caregivers of children attending preschools in the Caroni Education District.

Letters were sent to three preschools from a list used for a previous epidemiological study in the area, explaining the nature and purpose of the study. These letters were addressed to the head-teachers inviting them to have their school take part. Upon agreement, they were given letters to send to parents individually inviting them to be a member of a focus group and explaining what this would entail. When enough parents had responded, ideally four to eight participants per group, final arrangements were made to run the group session. In each school one teacher assisted in the organising of the focus group meeting with respect to co-ordinating the time, venue and requirements of the study investigators and liaising with the parents.

### Approval and consent

Individual written consent was obtained from all participants in the study.

The study had received ethical approval from The University of the West Indies, Faculty Research Ethics Committee.

### Conduct of the focus groups

The focus groups were run by a facilitator trained in the use of focus group methodology. The specific areas of interest for the discussion were introduced using a semi-structured topic guide (Table 1). This was a short set of pre-selected topics that comprised open-ended questions based on previous work with similar groups in other countries [23,24,29,30]. These questions were revised so that they would be culturally compatible and reflect the Trinidadian context. This was achieved through discussion between the researchers and from views of teachers/staff in the chosen preschools who were shown the topic guide. Questions were open-ended and probing, using terms such as ‘who’, ‘what’, ‘when’, ‘how’ and ‘why’. This guide was flexible and during the course of the discussions, not always asked in the same order and modified when needed, to explore emerging themes in greater depth. The focus groups lasted 45 to 50 minutes. The sessions took place in a quiet area of the preschools after the school day. During and after the session, light refreshments were made available to the participants to encourage a relaxed atmosphere and put participants at ease. Small gift bags (oral hygiene product samples, pamphlets and tokens), were also handed to each participants after the sessions as a courtesy for giving their time. 

**Table 1:** Focus group topic guide

	**Topic guide**
•	What does it mean to you to have a healthy mouth?
•	What are the kind of problems people might get with their mouths?
•	What are some of your experiences of going to the dentist?
•	Would you say that these experiences have affected you taking your child for dental care?
•	What would be the reasons for taking or not taking your child for dental care?
•	Which health professional would you feel most comfortable with for getting a dental check-up for your child?
•	How do you feel about your child’s first set of teeth?
•	How important do you think they are and why?
•	When do you feel is the best time to take your child to the dentist for the first time?
•	Are there things you know about that can prevent your child getting cavities?
•	How easy or difficult to do you find it to have your child brush their teeth?
•	Are there foods or drinks that you know may be good or bad for your child’s teeth?
•	Who makes the decisions about your child’s dental care?
•	Do other people (friends or family) influence these decisions?
•	What things would make going for dental care for your child easier or more comfortable?
•	What would make it easier for you to look after your child’s teeth?

During the group session, participants were encouraged to respond to each others’ comments and engage in an open discussion rather than just talk to the facilitator. As well as shared perspectives and consensus, unusual experiences and differing viewpoints on topics were welcomed, to gain a full range of opinions, feelings and insights. The discussions were recorded using a digital audio recording device and an audio cassette recorder. ‘Back-up’ field notes (including non-verbal communication), were also made by an assistant researcher who was present for all the discussions but took no part in them. Data were collected from 3 focus groups, in three different preschools between November 2010 and January 2011.

### Data analysis

A thematic content analysis was used to analyse the data. This was an iterative process that involved several readings of the verbatim transcripts, field notes and listening to the audio recordings. Initial analysis was based around the discussion topics used in the question guide (these having been developed from earlier studies on similar population groups).

Key early themes (proto-themes) that emerged from the transcripts were labelled and coded. These proto-themes were flexible categories which, following further study of the transcripts, were expanded and modified to encompass the issues that arose from within the data upon comparison of segments of text. These initial codes were developed with an assistant (RB) who also took field notes and checked the transcription. The codes were marked on the margins of the transcripts against corresponding areas of text, to enable retrieval and further sorting of the text.

Emerging themes were reviewed by an academic anthropologist (MF) at the University of the West Indies. Using ‘MS Word’ software, further refinement of the themes was achieved by placing segments of similarly coded text next to each other to explore deeper meaning and context. Final themes were developed and reported with a description and related quotations to underline their meaning.

### Respondent validity

The subjects were invited to comment on the focus group discussion (off-record) to consider whether they felt that it covered important/relevant issues and if it had fairly represented their feelings and thoughts. The participants of all three groups agreed that this had been the case.

### Reflexivity

The facilitator of the three focus groups was an academic dentist (RN) working in a teaching hospital and this was known to the participants. This did not appear to compromise the discussion and in some respects enabled participants to express concerns in detail, with respect to issues of access and barriers to care.

## Results

### Socio-demographic information

A total of 18 parents and caregivers participated across the three focus groups (FG1, FG2, FG3) with 5, 9 and 14 in each group respectively. These participants were mainly female (89%) with a mean age of 28 (ranging from 23 to 49 years-old). Just over two thirds (67%) were of Indian ethnicity, with the others being of African (28%) or Mixed ethnicity (5%). The majority were in manual/semi-skilled occupations or housewives, 33% and 39% respectively. Twenty eight per cent were in non-manual or professional occupations.

### Perceptions of oral health

Participants had reasonably clear thoughts on what constituted good oral health, with some expressing the view that this would include healthy gums and good breath and no cavities. Along with absence of disease, some felt that good oral health included having a nice appearance that aided self confidence:

"*Plenty of clean looking teeth*. (FG3)"

"*Gums that are not bleeding*. (FG3)"

"*Well umm… people with healthy teeth have no cavities and umm good breath*. (FG2)"

"*Helps your confidence in umm interacting with people.* (FG2)"

Pain and reduced function were mentioned by participants when describing an unhealthy mouth and not paying attention to oral hygiene could cause this. Also some felt that not taking good care of the teeth when young and heeding preventive advice could result in poor oral health later in life:

"*You wouldn’t be able to eat properly, with pain from sensitive teeth.* (FG3)"

"*Being able to chew…*"

"*Yes, you wouldn’t be able to chew or bite*. (FG3)"

### Dental care experiences

When discussing reasons for attending dental care, a recurring issue raised was that dental treatment was often unpleasant and that going to the dentist was something largely to be avoided, unless there was current pain or problems. However, some believed that this irregular attendance would result in more problems and were concerned by this happening to their child. Reasons for avoiding dental care were largely due to bad experiences, often during childhood. Fear was often related to the procedures, in particular dental extractions:

"*Toothache for sure…if you have a little cavity or so and it doesn’t trouble you then you find you wouldn’t really take care of it until you start to get pain, then you know okay, yeah you ready to reach to a dentist.* (FG1)"

"*it’s only when problems come then you well in my case that’s only when you know, have a problem then you would find yourself going to the dentist and you of course don’t want that for your children you want them to do the right thing carry them to the dentist.* (FG2)"

The environment of the dental clinic, that is, the smells, sights and sounds of the equipment and procedures were common issues of concern together with the chair-side manner of the dentist. Fear of reprimand for not doing a better job of looking after their mouth was also mentioned. Some participants described putting off taking their children for dental care due to their own negative experiences.

"*…I took too long to take them….and it’s because of my own experience, the trauma that you’ve been through, you know as children going to a dentist, I mean, so I transferred that.* (FG2)"

"*big as I am, I would really have to make up my mind to go to that dentist. If I don’t have pain, I not going for anything. Just to hear the machines, you know that ringing, buzzing thing in your ear.* (FG1)"

"*they will always find something wrong to tell you. You need a filling, you not flossing, you always, so you know, its not like you have any confidence going. you going because the dentist have something to tell you not doing good enough.* (FG2)"

Differences between private and public dental care were mentioned by several participants, who related this to their personal experiences and reasons for making choices as to which service to attend. In particular, some felt that private dental care would be safer and more comfortable for their child so having to pay for that was a worthwhile expense.

"*I prefer to pay your money and leaving it as that. You pay your money, you get your comfort, I believe in that.* (FG1)"

"*I am sure they have good workers (in government clinics). I mean they are all qualified people but umm the little extra to put out you know yeah, the little extra care, the little extra smile.* (FG2)"

"*You would pay so much just for somebody to be nice to you.* (FG3)"

"*to extract a tooth for your little one and there’s a needle going into the gum to numb the gum, you want that somebody who will take that extra care with your little one, that’s your little one you know.* (FG2)"

"*the way they going to treat your children, the way they going to treat you when you reach you know, I think, you know, hear what, I may be safer by a private dentist.* (FG2)"

Some participants felt that private dental practitioners were aware of the lack of services available in the public sector and tended to exploit the situation and also felt they had little information of the government services available:

"*I guess the dentist knows that there is no other reputable treatment available for children or otherwise, so they make their money.* (FG1)"

"*could sometimes be unfair too. the professionals know that the health service is bad whether it be dentistry, maternity whatever so you know what. they know you’re gonna pay for it. So they call their price and they will get their patient.* (FG2)"

"*and I also didn’t know about the Government.*"

"*I didn’t know, cause if I knew that, I think I would have taken my children there.* (FG1)"

### Dental care for children

Participants were clear in knowing what they would like the dental visit for their child to be like. The personality of the dental care provider was the focus, with several stating that they would want someone who could put the child and ease and communicate well with them. The environment of the clinic was important to most participants and making it less intimidating and ‘child-friendly’ by reducing the clinical sounds and smells and having tokens and rewards for the child was seen as very helpful.

"*Somebody pleasant who would make them feel comfortable and not amm not put them down too much even though they have cavities, you know explain to them how they get the cavities but how they could also help prevent it you know and take care of their teeth, some encouragement you know.* (FG1)"

"*A room that is ..not scary...not too sterile, a room that is fun like ..* (FG2)"

"*It could be sterile clean but not smell you know the scary smell that you get in the hospital…there should be some brightly coloured pictures* (FG2) *they should never ever hear that…’.zzzzzzzzz’…that sound, that sound alone is scary* (FG2)"

"*Friendly, yes, friendly person…not just come out and say ‘Your turn’ you know.*"

"*Somebody to talk to the child . talk to the child and be friendly* (FG3*)*"

### *The first dental visit*

There was not much awareness of when to take a child for the first dental visit (other than if there were pain or problems in need of attention). Getting them checked around the time the child would have a full set of primary teeth or preschool age seemed to be the general view:

"*I’m going to take her for the first dental check-up, she will be four. I don’t know if that is right but you know.* (FG2)"

"*My son was probably about five or six, but they didn’t really do anything major, they just looked at his mouth.* (FG3)"

"*My dentist is so warm and so nice that I even told her okay my baby girl is going to come here, right because next month she is going to be four you know you tell me when, what age is good, and I want her to come* (FG1)"

### Importance of “baby” teeth

There were mixed opinions about the importance of the primary teeth. Several participants felt that as the teeth were temporary they were not too concerned about them getting cavities and would focus more on the permanent teeth once they came in. However others believed that problems with the primary teeth could affect the permanent successors and were worried about decay or infections causing pain and problems.

Although aware of their temporary nature, some participants felt that the primary teeth could help the child to enjoy their food allowing variety in their diet and also provide an opportunity to learn good oral hygiene habits.

"*I not too worried about because I know the teeth will fall out as is baby teeth and they would get the adult teeth and you know.* (FG1)"

"*I don’t think they so important, because I find they just drop off then you get adult teeth you know.* (FG2)"

"*I feel I have more concern about the permanent teeth.well I mean after that baby teeth come out it’s the permanent teeth coming in and when that come out no other teeth coming in so you wanna protect them.* (FG3)"

"*Well I think if this teeth, the first set of teeth bad, it will affect the next one not so, the new ones? That is what I always thought it was like that.* (FG3)"

"*is like their trial teeth you teach them to brush and take care of it you know and show them if they get that little bit of pain in that set, you could imagine in the permanent set how much more the pain could be.* (FG3)"

### Diet and food choices

With respect to healthy diets for their children, participants were aware of the risk posed by sweets and chocolates but also conscious of providing a generally nutritious diet. In terms of preventing cavities, more emphasis was given to brushing than the diet.

"*you have to kind of teach them from early and groom them into what you want them to get into, good eating habits, good hygiene, you know you start them from now*. (FG3)"

"*they have to eat everything to get all the proteins and calcium and all these things, its just to brush.* (FG1)"

"*I think brushing is more important*. (FG1)"

### Use of the bottle and breast feeding

Participants felt bottle feeding before bed-time had a role to play in providing comfort for the child and aided in getting them to fall asleep. During the day bottle feeding would allow housework or other activities to get done by the caregiver. There was a generally good level of awareness that falling asleep with the bottle and not brushing the teeth at night might result in cavities, with advice on this received from dental professionals. Some participants felt the child took more food via the bottle rather than the cup and being concerned about overall nutrition, favoured continuing that approach. Breast feeding was seen as beneficial by most of the participants and several had continued into preschool age. The issue of breast milk causing tooth decay was known but caused considerable confusion as several participants had received conflicting messages from dental and medical professionals:

"*One thing about the bottle feeding, it is a source of comfort for the child that they find, you know they even fall asleep with the bottle in their mouth…* (FG1)"

"*I think it’s a concern because parents sometimes doing their chores and sometimes just for them to do their chores at home and to make baby comfortable, same time they give them a bottle and sometimes they fall asleep with the bottle in their mouth.* (FG1)"

"*Yes, well I give him in a cup nah because he drinks from the cup. just he drinks more if he drinks from the bottle*. (FG3)"

"*Yes, a dentist told me that, so I was shocked* (breast feeding can cause cavities) *and the doctor saying breast is the best thing*. (FG2)"

### Tooth brushing

The importance of tooth brushing for their children was appreciated by many of the participants, particularly brushing last thing before bedtime. Concerns about leaving sticky sweet foods or milk on the teeth during the night was understood to be a risk for cavities. Difficulty in achieving night-time brushing was often due to the child falling asleep soon after the last meal.

Supervision of brushing was seen as important due to an understanding that the child lacked manual dexterity and brushing on their own would be somewhat ineffective. However, some participants mentioned that they wanted to encourage the child and build their confidence and not make them feel that they couldn’t do it for themselves, so they would let them brush their own teeth.

"*Especially your child brushing before you go to sleep is important because you have that chocolate and things stuck in your teeth overnight given that time the cavities form and whatever*. (FG2)"

"*brushing is a big thing too. The night brushing …. they kind of now starting it, they do it now and then I have to be there.*"

"*Umm…but I am trying to get them to do it now on a daily basis the night brushing*. (FG1)"

"*That was the downfall not brushing in the night…you go to the dentist and he said make sure and get him to brush his teeth, when he drink his tea let him stay up and go to brush his teeth before he go to bed*. (FG3)"

"*She wouldn’t want me to help her because she would think that she’s not brushing clean enough.* (FG2)"

### Toothpaste and concerns with fluoride

Considerable confusion was evident among participants around the issues of fluoride and its role in dental health. Being unable to get clear information and advice on its use in young children was apparent. Some were not using fluoride toothpaste due to concerns about young children swallowing excess paste and its toxic effect on their bones. Articles seen on the internet, news and published media about harmful effects were a general concern. Some participants were using non-fluoride / herbal toothpaste for themselves and the availability of non- or low-fluoride children’s toothpaste, from established brands, seem to confirm fears of using fluoride toothpaste for their children.

"*You know you see a lot of advertisements that fluoride toothpastes are not good for small children because they swallow it, they say its not good for their bone development. I want to know if that is true because I still use a non-fluoride toothpaste*. (FG1)"

"*….I have switched… (XXXX) bring out their new toothpaste, a non-fluoride baby toothpaste. This is from ages zero to two, they normally recommend that for children who can’t spit yet, well at least I think so, but I still give him the non-fluoride*. (FG1)"

"*Yes, on the internet you see its bad.*"

"*I don’t want to use it, I use a herbal one*. (FG2)"

"*It was on the news, on the news and alternative books also talk about fluoride, how bad fluoride is, you know alternative medicine and all that..* (FG2)"

### Influence of family and friends

A common issue among the participants was the lack of control, bordering on frustration over their child’s diet, due to other family influences and environments outside of the home where snacking on sweets / sodas was encouraged. On the whole, the giving of sweets by grandparents and family was acknowledged as an expression of caring, however, participants felt pressured by their child demanding sweet snacks from them because of having seen their friends at school or in the neighbourhood getting them. Keeping sweets snacks in the home was also problematic as children would not be able to self-limit:

"*Grandparents say- when you were small you use to eat lollipop too and why can’t they have it now* (FG1) *the Grandparents don’t have a problem giving her soft-drink but I have a problem giving soft-drink* (FG2) *when the children live like with grandparents they would have a greater influence than the parents who live by themselves you know, when you brush teeth, is either morning or night so the Grandparents if they don’t really live with you they don’t really have that impact*. (FG2)"

"*It’s a bit difficult, like if you go anywhere and they see you know like if you go to somebody house and they have like the chubby and snacks and stuff like that, Mummy I want, and when you tell them no you can’t get that, you know they still want it*. (FG3)"

### Sources of dental health information

Information on dental care for young children was sought from a variety of sources. Those who had contact with dental professionals were able to obtain it more easily whereas others relied on friends and family. Dental care information during the perinatal period and infancy was particularly difficult to access even in the nursing home environment. Participants appeared to trust government sources but felt that much more could be done to help educate them via written material or the broadcast media.

"*So you’re kind of working on your own wondering like okay when is it good to start brushing ….what type of toothbrush to use, what kind of toothpaste*. (FG2)"

"*Yeah you’re kind of on your own and your probably asking you know friends who have kids or whatever*. (FG2)"

"*We did not always have the internet so easily accessible for everybody so you depend on these reading materials.* (FG1)"

"*Yeah hopefully it would be from a reliable source whether it be government institution you know the Ministry of Health you would feel comfortable.* (FG2)"

"*Even brochures, when you go to the clinic (health centre), you can always find ones on diabetes or hypertension things you can sense before. Yes they have them, so brochures on dental health would be nice.* (FG2)"

### Main caregiver responsibilities

Making decisions about dental care for the child was largely seen as the mother’s responsibility with male partners not sharing this role equally. Mothers felt under pressure to control food choices and implement preventive approaches to dental care in addition to the general demands of childrearing and home care.

"*It’s mummy who have the problem……Mummy who have to wake up whole night*. (FG1)"

"*Yeah I have the same thing. I make most of the decisions concerning my sons, their daddy he’s like a chocolate and a ice-cream freak*. (FG3)"

"*No we are the ones who have to call and say ok you have to go for a check up…* (FG2)"

"*I think my son’s father is concerned about the children’s teeth, but he does be like ‘You brush this child teeth yet? I’m like ‘No, why you don’t go and brush it’*. (FG3)"

"*amm. think not being able to control my children to tell them not to eat the sweets, and you feel terrible, imagine that you can’t control your child from eating the sweets. We are the adult, they are the child, it means, you are not in control of. That’s, that’s, that’s what is hard. So you know, you kind of go on a guilt trip, you know, okay. It’s in the choices that we make you know.* (FG2)"

### Suggestions to improve dental care for young children

For them to maintain their children’s oral health, the participants felt that better access to dental services, largely through reducing the cost factor would help them. More mention was made of having more readily available information and education, both through written material and face-to-face scenarios with dental professionals, such as workshops and programmes in schools via the parent/teacher networks. It was felt that the Ministry of Health could do more to support these kinds of activities.

"*Check-ups so that your child is monitored throughout and then also a less prohibited cost when doing so because we want to do it but because of the cost we can’t do it*. (FG1)"

"*.a dentist or you know. somebody who comes with some information to the schools and let the teachers know*. (FG3)"

"*Having a Parent Teacher Meeting, let somebody come and advise them listen you know you have to help at the school as well as the parents have to help at home.* (FG3)"

"*workshops….so even though you have a pamphlet to read and something have the professional to ask and see if I really understand, if I am doing the right thing you know whatever questions you want to ask you could ask.* (FG3)"

"*you know if we have to start taking care of children’s teeth as early as this age then we need to get help and information from this age or at least earlier to be prepared.* (FG3)"

"*Well I would like if amm the Ministry of Health could get some more workshops in the schools for parents, let different dentists come in….you know meet parents give us some sort of information where we could help our children from early to take care of their teeth to take better care*. (FG1)"

## Discussion

The majority of caregivers in this study were female, in manual work or housewives, in their mid to late thirties and predominantly of Indian ethnicity. The ethnic composition reflects that of the county of Caroni, a former agricultural area and home to the majority of indentured labourers brought from India to Trinidad, during British colonial rule, to work on the sugar estates. The older than expected age of the group may reflect the fact that several of the participants were not first time parents and cared for older children along with those of preschool age. Although not formally recorded, several participants appeared to care for more than one infant or preschool child.

As reported in the results the three focus groups yielded considerable comment and discussion. The following list provides an overview of the main findings:

Participants generally valued good oral health.

Negative dental experiences were common and affected dental attendance.

Difficulty in accessing dental care for young children was common due to issues of affordability and availability.

Ambivalence toward the role of primary teeth.

Confusion over the role of fluoride and timing of child’s first dental visit.

Bottle-feeding was seen to play a role in caring for the preschool child.

Difficulty in managing external influences when trying to control child’s exposure to sweet snacks and drinks.

Toothbrushing and oral hygiene were felt to be more important than diet in preventing decay.

Problems with accessing oral healthcare information and a need for more practical advice.

Participants’ views of oral health in the main referred to a mouth free from pain and cavities, clean teeth, and healthy gums that did not bleed. Along with these issues were aspects of function and aesthetics. In particular, being able to chew and eat comfortably and having a nice smile that would allow one to socialise and make a good impression, were highlighted. Good oral health therefore appeared to be valued and contributed to quality of life, which is similar to findings from other adult populations where most people felt oral health affected their social and psychological well-being [31,32].

Views of oral health are known to be influenced by social and cultural norms [33]. The paucity of data on this issue in the Caribbean suggests the majority of adults perceive their oral health as very important to them [34] which is supported by the findings of this present study. Negative views of oral health or oral health fatalism (i.e. belief that poor oral health was inevitable and cannot be avoided), did not appear to be a major factor, as participants in the present study felt that taking preventive action such as having dental check-ups and following advice on care of the mouth would help avoid future problems.

Several participants recalled unpleasant experiences during dental treatment that they had received in the past, which is consistent with other, similar qualitative studies [23,25,26] some also admitted transferring their dental anxiety to their child and this is likely to have affected their attitude toward dental care for children. In some cases, these were events that occurred during their own childhood. Mothers’ fear of dental treatment is known to be a factor influencing routine dental care for their children [35]. Mothers of preschool children in the UK [25], reported that dental anxiety, along with cost of treatment were their main barriers to seeking dental care.

Some participants in this Trinidadian study also felt an element of guilt in not being more proactive in seeking dental care for their child due to these negative perceptions of dental care, again similar to views expressed by mothers in the UK [36]. Interestingly, even where a child has had a recent good dental care experience, the parent may still be unable to separate between their past bad experiences and their child’s potentially positive one [21,23]. Based on personal experiences of dental care, most expectant mothers in the US would not seek dental even if they had a problem [26] and were anxious about what would happen to their child if they took them to a dentist.

Fear of dental local anaesthetic (LA) injections and having teeth extracted were commonly cited by participants in the present study, which is similar to findings from a previous study of Trinidadian adults attending a dental hospital clinic, where over two thirds of the sample had avoided dental treatment in the past because they were too anxious and over half still had anxiety about LA injections or extractions [37]. This is therefore an important public health issue, as dental anxiety can result in worse oral health, compared to dental patients who are not anxious. For instance, people highly anxious of dental care have been found to have more missing teeth, fewer filled teeth and more likely to be in need of urgent care [38,39].

The general opinion in the present study was that better quality care was available for those who could afford it and some indicated that they would seek private dental care for their children in order to make the visit as comfortable as possible even if it involved considerable financial sacrifice. There was a certain level of acceptance that this was just part of the norm in health-care in Trinidad and a reflection of an established ‘two-tier’ health system. There was also a general feeling that government dental services e.g. health centres and the Dental Hospital, were not well advertised and information on location and services offered were hard to come by, which increased the barriers to dental care for the children. Some participants also believed that dentists were aware of this situation but unwilling to challenge it, as it resulted in more people having to seek private care suggesting a degree of mistrust of the system.

Invasive dental treatment of preschool age children can sometimes be challenging for both patient (their caregiver) and the clinician. Pine [40] reported that young children not being able to cope with dental treatment was one of the main barriers to care contributing to health inequalities in childhood dental caries.

Most comments about making the child’s dental visit pleasant and productive related to the clinic having a non-threatening atmosphere. This included not having a hospital smell and image but rather a colourful and happy environment, ideally with the child receiving toys and tokens (stickers / toothbrush etc.) after a visit. The dentist having a friendly and warm personality and able to put the child at ease, was also highly valued. These findings echo those of Finch [41] where similar aspects of the dental visit and the personality of the dentist were some of the main barriers to care for adults in the UK and in an ethnographic study of parents of preschool children in rural California there was general dissatisfaction with what they perceived as an insensitive attitude of dentists in the management of children who were anxious and/or uncooperative during treatment [42].

The importance of the primary teeth and timing of the child’s first dental check-up suggested there was considerable confusion among the participants on this issue. Consistent with other similar studies a view that the ‘baby teeth’ were temporary and hence not vital to maintain was common [23,24,29]. Participants were somewhat unsure of the role the primary teeth play in oral health and the development of the permanent dentition. Similar to caregivers in the US [24], there were a variety of views as to when to take a child for their first dental check-up. Most felt that this would be around the time the child had all the primary teeth, about age three to four years. This is of concern as both the British Society for Paediatric Dentistry (BSPD) and the American Academy of Paediatric Dentistry [43,44] state that dental attendance should ideally occur by the time the first teeth erupt or by one year of age. Early dental attendance can also enable the delivery of anticipatory guidance from a dental health professional about the child’s growth and development, preventive dental care and the establishment of a ‘dental home’ [45]. Early dental attendance has also been shown to increase the likelihood of being caries-free later in childhood [46].

Participants were aware of the role of sugary foods and drinks in the child’s diet and the importance of trying to limit their intake to prevent dental caries. Participants seemed aware of the issue of giving a child a healthy and balanced diet which included fruits and vegetables. Cultural factors may play a role in the giving of sweets snacks and confectionary. This seems very much part of normal family life and social interactions in the Caribbean, there being something of a ‘positive value’ ascribed to sweet foods. In this respect the availability of sweet snacks and drinks provided by grandparents, other family members, neighbours and school parties was also seen as quite challenging. This was similar to mothers in the UK who felt pressured when their child wanted snacks they had seen other children have [25]. The pervasive availability of sweets and sugary snacks also presented difficulty for parents and caregivers despite their being aware of the importance of good dietary habits for their children, a finding also reported in the US [24]. Regularly receiving sweets snacks and confectionary from grandparents was a common finding among preschool children undergoing caries related tooth extractions in the UK [47].

Participants in the present study also connected bottle-feeding and breastfeeding to good nutrition, as well as being a comforting and practical part of childcare. Some therefore found weaning from the bottle quite difficult, similar to US caregivers [24],

More emphasis was placed on diet compared to oral hygiene, as tooth-brushing habits were relatively easier to establish but these parents were already part of a health promotion programme (Sure Start), in the UK [25]. Similarly diet, more than lack of brushing, was perceived to be the main cause of caries in a qualitative study health professionals (dental and non-dental), who had early contact with mothers of preschool children in Australia [22]. The present findings also differ somewhat from those from parents of preschool-aged children attending a dental hospital clinic In Trinidad, who had lower awareness and some confusion regarding the importance of oral hygiene and other caries preventive measures for their children [17].

Oral health behaviours and parental involvement in children’s oral health has been linked to the concept of self-efficacy. Self-efficacy refers to a person’s conviction that they can successfully execute the behaviour required to produce the desired outcomes [48] that is the belief in his or her ability to carry out and succeed at a specific task [49]. In an investigation of the relationship between ECC with familial and cultural perceptions and beliefs in a 17 - country study, parental self-efficacy was the strongest predictor in the establishing of tooth-brushing habits and controlling sugar snacking [15] Maternal oral health self-efficacy (OHSE) was also strongly related to tooth brushing frequency in a study of African-American mothers of 1–5 year-olds in low income families [16]and mothers who had more knowledge about their children’s oral hygiene needs felt more efficacious, indicating the importance of imparting oral health information early in the life of the child.

There was a surprisingly high level of concern and confusion as to the value of fluoride and its safety in young children, similar to previous a study in Trinidad [17] and to some data in the US [26]. It is therefore imperative that information about fluoride needs to be imparted to parents and caregivers in a clear and accessible medium, to counter negative perceptions and fears and to support self-efficacy. Anticipatory guidance must include the message that children should have optimal exposure to fluoride to prevent caries as Adair *et al.*[15] state “children are more likely to be caries-free if their teeth are brushed twice daily with fluoride toothpaste, with parental involvement and in an environment where sugar is controlled”. Furthermore, there is no clear evidence that toothpastes containing less than the standard concentration of 1000 ppm are effective in preventing caries in young children and hence low concentrations are no longer advised for preschool children and infants [50]. Avoiding fluorosis in the permanent dentition, due to over-ingestion of fluoride is however, an important issue. Current evidence-based guidelines recommend only a ‘smear’ of toothpaste on a small toothbrush for a child up to age 3 years, covering no more than three quarters of the brush head and a ‘pea size’ amount for children up to the age of 7 [51].

Most participants appeared to rely heavily on family and friends as sources of health information, including advice for children’s oral health. These traditional social networks seem to have compensated for what appears to be a lack of information from more ‘official’ sources locally. Some of these findings also relate to the issue of ‘health literacy’. The WHO describes health literacy as “the cognitive and social skills which determines motivation and ability to access and understand health information” [52]. Health literacy is critical to empowering people to promote and improve their health [53] as it can influence access to health information, managing personal health, those cared for and utilisation of health services [54]. This also extends to oral health, with ‘oral health literacy’ defined as *‘the degree to which individuals have the capacity to obtain, process and understand basic health information and services needed to make appropriate oral health decisions’*[53]. In the present study, the request for more written material suggests that level of health literacy among the participants was quite high, hence more information on dental services may improve uptake and preventive care. This is encouraging but may not be representative of communities where oral health literacy is lower, so other forms of imparting information on early childhood oral health would need to be considered, such as face-to-face delivery through talks and workshops etc. Lack of daily tooth brushing and night-time bottle use were associated with lower health literacy among female caregivers in the US and appears to be independent of SES factors, including education [55].

Despite some ambivalence about the role of the primary teeth, most participants understood the need for maintaining good oral health for their preschool aged children, the importance of healthy nutrition and oral hygiene. However, similar to Daly [25], they seem to want more practical help in how to translate this knowledge into effective dental health care on a day-to day basis for their children.

### Recommendations

Participants wanted clear and explicit information on brushing, use of fluoride and when best to take a child for a dental examination. These issues are in fact at the core of dental health educational messages for preschool children and therefore need more effective dissemination and delivery in Trinidad.

Dental health education in the preschool and primary school setting could be considered. Encouragingly, primary school teachers in Trinidad have shown positive attitudes towards including dental health in the school curriculum, provided they receive support and training [56]. Other dental public health strategies that should be considered include anticipatory guidance and motivational interviewing. These counselling-based approaches can encourage early dental attendance (e.g. age 1 dental visit), establishment of a dental home and appropriate use of fluoride, which can reduce risk of ECC [57,58].

Increasing the availability of dental care for children needs to be addressed as problems with access to care were common. This may be possible with increased training and deployment of Dental Nurses/ Therapists in the country. These mid-level dental providers in Trinidad have previously indicated enthusiasm for providing dental health education and out-of-clinic interaction with young children [59] and globally been shown to be effective in increasing access to care for children [60]. Improving early childhood oral health and prevention of ECC in Trinidad should involve discussion of research findings and potential public health strategies with key stakeholders such as parents, caregivers, teachers, health professionals and policy makers, to enable delivery of appropriateness interventions.

### Limitations of the study

As the qualitative method used was not designed to produce data to be extrapolated to the population, a limitation might be whether these findings represent the average views of parents and caregivers in Trinidad. The participants in this study came from an ethnically diverse, mixed urban /rural community which is common to most of the island. The views shared by them are therefore likely to be relevant to parents and caregivers across Trinidad.

Although all three groups received all the questions on the topic guide, some received it in a slightly different order. Though this was done with the aim of being responsive to the participant’s views and encourage the flow of the discussion, a possibility of bias may have arisen from not standardizing the sequence of questions. The order of questions may have influenced some responses, for instance asking about the ‘importance of baby teeth’, after asking the question on ‘reasons for not taking a child for dental care’ might have produced a different response if asked in the reverse order. Also no specific question was asked about dental visit by one year of age.

Some debate exists around minimum sample sizes required in qualitative studies, with respect to achieving data saturation. A minimum of 15 has been cited [61] and Guest [62] states that where there is a high degree of homogeneity in the study population, data saturation can occur with very small samples (less than 10), with data quality being measured by value rather than sample size. In the present study, the main themes described arose in all three focus groups suggesting that these were relevant and important issues for these participants.

Since the facilitator was a dentist, this might have inhibited some disclosure on personal experiences of dental care or views of dentistry, yet the perception was that the discussions appeared honest and uninhibited.

## Conclusion

This qualitative study provides the first information of its kind on the experiences of parents and caregivers with respect to oral health of preschool children in Trinidad and reveals some important issues for the development of oral health promotion strategies.

The participants showed generally positive attitudes towards oral health but appear to have encountered several barriers and challenges to achieving ideal preventive care for their child, with respect to maintaining a healthy diet, good oral, hygiene and dental attendance. Dental attendance for young children appeared to be influenced by participants own negative dental experiences. Oral health promotion should include effective dissemination of oral health information, more practical assistance and greater access to dental care for families with preschool children.

## Abbreviations

ECC: Early childhood caries; OHSE: Oral health self efficacy; AAPD: American academy of paediatric dentistry; BSPD: British society of paediatric dentistry; WHO: World health organisation.

## Competing interests

The authors declare that they have no competing interests in this research. No financial or non-financial interests influenced the interpretation of the data or presentation of the information.

## Authors’ contributions

RN conducted the fieldwork and data acquisition. RN, JN and MF contributed to the design of the study, data analysis / interpretation and preparation of the manuscript. All authors read and approved the final manuscript.

## Pre-publication history

The pre-publication history for this paper can be accessed here:

http://www.biomedcentral.com/1472-6831/12/27/prepub
